# Special Staining and Protein Expression of VEGF/EGFR and P53/NF-κB in Cryptorchid Tissue of Erhualian Pigs

**DOI:** 10.3390/life14010100

**Published:** 2024-01-08

**Authors:** Penggang Liu, Yiming Shao, Caihong Liu, Xiaoyang Lv, Seth Yaw Afedo, Wenbin Bao

**Affiliations:** 1 College of Veterinary Medicine, Yangzhou University, Yangzhou 225009, China; 2 International Joint Research Laboratory in Universities of Jiangsu Province of China for Domestic Animal Germplasm Resources and Genetic Improvement, Yangzhou University, Yangzhou 225009, China; 3 Joint International Research Laboratory of Agriculture and Agri-Product Safety of Ministry of Education of China, Yangzhou University, Yangzhou 225009, China; 4 Department of Animal Science, School of Agriculture, University of Cape Coast, Cape Coast P.O. Box 5007, Ghana; 5 Key Laboratory for Animal Genetics, Breeding, Reproduction and Molecular Design of Jiangsu Province, College of Animal Science and Technology, Yangzhou University, Yangzhou 225009, China

**Keywords:** Erhualian pigs, testis, VEGF/EGFR/P53/NF-κB, expression, histology

## Abstract

Erhualian pigs exhibit one of the highest reproductive rates globally, and cryptorchidism is a crucial factor affecting reproductive abilities of boars. This investigation focused on cryptorchid tissues from Erhualian pigs, where the histological structure of cryptorchidism was observed using specialized staining. In addition, protein expression of P53/NF-κB in cryptorchid tissues was assessed using Western blot and immunohistochemistry. In comparison to normal Erhualian testes, Masson’s trichrome staining indicated a reduction in collagen fibers in the connective tissue and around the basal membrane of the seminiferous tubules in cryptorchid testes. Moreover, collagen fiber distribution was observed to be disordered. Verhoeff Van Gieson (EVG) and argyrophilic staining demonstrated brownish-black granular nucleoli organized regions in mesenchymal cells and germ cells. When compared to normal testicles, the convoluted seminiferous tubules of cryptorchids exhibited a significantly reduced number and diameter (*p* < 0.01). Notably, VEGF/EGFR and P53/NF-κB expression in cryptorchidism significantly differed from that in normal testes. In particular, the expression of VEGF and P53 in cryptorchid tissues was significantly higher than that in normal testes tissues, whereas the expression of EGFR in cryptorchid tissues was significantly lower than that in normal testes tissues (all *p* < 0.01). NF-κB expressed no difference in both conditions. The expressions of VEGF and NF-κB were observed in the cytoplasm of testicular Leydig cells and spermatogenic cells, but they were weak in the nucleus. EGFR and P53 were more positively expressed in the cytoplasm of these cells, with no positive expression in the nucleus. Conclusion: There were changes in the tissue morphology and structure of the cryptorchid testis, coupled with abnormally high expression of VEGF and P53 proteins in Erhualian pigs. We speculate that this may be an important limiting factor to fecundity during cryptorchidism.

## 1. Introduction

The reproductive ability of Erhualian pigs, recognized as one of the pig breeds with the largest litter size globally, has attracted scholarly research and attention. In recent years, a substantial decline in the numbers of various pig breeds has been attributed to diseases and inadequate breeding conservation. This decline not only constitutes a severe setback to pig breed resources but also presents a formidable challenge and threat to breed conservation and the selection of high-quality strains [1]. The testis is a pivotal organ for assessing boar fertility. However, testicular dysplasia is an important factor affecting the fertility of boars. Cryptorchidism, a prevalent manifestation of testicular dysplasia in both humans and animals, is reported at rates ranging from 2.5% to 4% in humans, 0.8% to 9.7% in dogs [2], 3% to 6.3% in cats, and 2% to 2.2% in pigs [3]. Studies indicate that the coordinated regulation of testicular development and spermatogenesis in postnatal porcine involves multiple signaling pathways, such as TGF-beta, PI3K-Akt, Wnt/β-catenin, and AMPK [4]. 

The overexpression and knockout of the vascular endothelial growth factor (VEGF) directly impact the proliferation and metabolism of testicular Leydig cells, which is pivotal in testosterone secretion and the regulation of testicular development and sperm maturation. For instance, the absence of the VEGFA subtype in Sertoli and germ cells disrupts amino acids crucial for the stable maintenance of undifferentiated spermatogonia, resulting in a decreased sperm count and reduced fertility [5]. VEGF nanoparticles can enhance the micro-environmental metabolism of new blood vessels, promoting blood vessel formation and preserving vessel wall integrity. In mouse cryptorchidism, VEGF expression decreases time-dependent growth, and this is consistent with histopathology and weight. Metabolic decline and apoptosis commence on the seventh day in spermatogenic cells of cryptorchid mice [6]. The VEGF/VEGFR2 axis may play a key role in the survival of endothelial cells in the varicocele vein by inhibiting apoptosis and stimulating angiogenesis. Furthermore, elevated VEGF levels in the testis might contribute to the suppression of spermatogenesis and infertility, which are associated with varicoceles [7]. Notably, VEGFA levels were significantly higher in the testicular fluid and serum of rats after an immunization period preceding testicular damage. VEGFR2 increased in the testicular interstitium after the immunization period. Our findings suggest that VEGFA could potentially serve as an early marker for testicular inflammation, subfertility, and infertility [8].

The epidermal growth factor receptor (EGFR) functions as a receptor for epithelial growth factor, participating in cellular processes, such as cell proliferation, angiogenesis, microenvironment metabolism, and cell apoptosis. Moreover, preliminary data indicate that in cryptorchidism, germ cell counts and estrogen receptor alpha (EGFRα) mRNA expression in the gubernaculum and epididymis are significantly reduced, while estrogen receptor alpha protein expression in the testis appears to increase, indicating aberrant regulation [9]. In cryptorchid boys, EGFR expression in testicular Leydig cells is lower in boys aged approximately 2 to 4 years than compared to those aged 5 years (*p* < 0.05). Therefore, EGFR expressions may correlate with age and cryptorchidism [10].

Recent studies reveal the involvement of P53 in the metabolism, proliferation, and generation of testicular Leydig cells and spermatogenic cells. The data emphasize molecular mechanisms underlying interactions among the Takeda G protein-coupled receptor 5 (TGR5) and tumor suppressor protein (TP53) pathways in spermatogonia associated with germ cell metabolism and apoptosis following busulfan exposure [11]. In cryptorchid testes, compared with normal testes, the mean weights of abdominal testes were significantly reduced at 7 and 14 days post-surgery, and this was accompanied by significantly increased apoptotic germ cells. A Western blot analysis indicated a significant upregulation of P53 expression in purified germ cells 7 days after surgery [12]. The enhancement of reproductive metabolism of obese mice, characterized by improved testis morphology, sperm parameters, acrosome reaction, and embryo quality after in vitro fertilization, was achieved via silent information regulator 1 (Sirt1) activation and p53 deacetylation [13]. The high expression of P53 in rat testicular tissue promoted apoptosis of spermatogenic cells, resulting in a significant decrease in spermatogenic cells and male sterility [14].

Herein, we demonstrate that the transmembrane receptor activator of the nuclear factor-ĸB (NF-Kb) ligand signaling system is active in the mouse testis [15]. NF-ĸB gene expression, when significantly reduced, improves semen quality. NF-κB demonstrates potent antiapoptotic effects with respect to AGE-induced GC-2 cell damage compared with loganin alone [16]. The abnormal expression of NF-κB in the undescended testis is evident, and it results in eventual abnormalities in male reproductive function [17]. Additionally, cryptorchidism induces the upregulation of NF-κB, resulting in the breakdown of testicular tight junctions and impaired function of supporting cells [18].

Despite these findings, there is currently a dearth of reports on VEGF/EGFR and P53/NF-κB in swine cryptorchidism. Consequently, we observed and compared histological characteristics of cryptorchidism to normal testis and analyzed the differential expression of factors related to testis metabolism and development. This study is a significant guide for understanding testicular development and sperm maturation in breeding boars.

## 2. Materials and Methods

### 2.1. Animals and Sampling

A total of 10 Erhualian pigs aged 28 to 35 days were studied, including 5 with cryptorchid and 5 with healthy testicular tissues. The materials of the present study were collected from the Erhualian pig breeding base in Jiangsu Province, China. This experiment was approved by the institutional review board of Animal Ethics Committee of Yangzhou University (SYXK(Su)2017-0044). Written informed consent was obtained from the owners for the participation of their animals in this study. All pigs were kept under same natural environment (altitude, approximately 50~150 m; temperature, 14~22 °C; and medial humidity, 63~84%). The experimental pigs were fed automatically in accordance with the operations of the mechanical automatic pig farm.

### 2.2. Materials

The tissue samples were surgically removed from cryptorchid and healthy Erhualian pigs and trimmed to a size of 1 cm^3^ and immersed in 4% paraformaldehyde solution for 12 h. The samples were dehydrated with ascending gradient alcohol (70%, 80%, 95%, 100%), xylene replaced anhydrous ethanol for transparency, tissues were penetrated with paraffin wax and embedded, and the paraffin tissue blocks were stored at 4 °C for follow-up histological experiments; protein detection samples were stored in the refrigerator at −80 °C. 

### 2.3. Preparation and Staining of Paraffin Sections of Testicular Tissue

For Masson staining (G1006, Servicebio, Wuhan, China), dewaxing of tissue sections was carried out with xylene, downward gradient alcohol dehydration (100%, 95%, 80%, 70%), ascending gradient alcohol dehydration, 2.5% potassium dichromate overnight, immersion of Weigert iron hematoxylin and ponceau acid fuchsin, 1% phosphomolybdic acid and 2.5% aniline blue solution staining, ascending gradient alcohol dehydration (70%, 80%, 95%, 100%) and xylene transparency, and neutral gum seal piece. For Verhoeff’s Van Gieson (EVG) staining (G1035, Servicebio, Wuhan, China), dewaxing to water steps were as above, with Verhoeff’s Van Gieson differentiation after staining, Van Gieson counterstaining and alcohol dehydration, and sealing for observation. For Argyrophilic staining (G1042, Servicebio, Wuhan, China), dewaxing to water steps were as above, with ultrapure water washing after acid formaldehyde, silver glycine staining, 45-degree preheated reduction solution for color development, and microscope observation of dewatered sealing.

### 2.4. Counting of the Number of Convoluted Tubules

**Figure 1:**
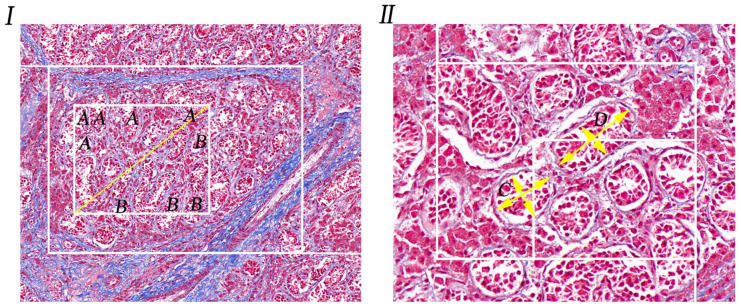
Diagram of measurement of number and diameter of convoluted tubules. (**I**). Masson’s trichrome stained of normal porcine testis, statistical curve tubule number pattern, 20×; A is the convoluted tubule included in the statistical results; B is the convoluted tubule not included in the statistical results. (**II**). Special stained section of pig cryptorchidism; schematic diagram for measuring the diameter of the tubule of the curved sperm, 40×; C and D were selected in order to measure the diameter of the curved tubules in this field of view, and the cross measurement method was adopted.

A number of uniformly distributed measuring fields of the same size were randomly selected from the testicular slices of pigs. A rectangular measuring frame was superimposed on each measuring field, and the profile of spermatogenic tubules located within the measuring frame was selected according to the forbidden line rule. Forbidden line rule is an unbiased method to count contours with the naked eye. Its principle is to count only the contours that are completely in the measuring frame or intersect only with the right or top of the measuring frame, as shown in Figure 1-I (A), but not the contours that intersect with the left, bottom or left and right side extension lines of the measuring frame, as shown in Figure 1-I (B) [19].

### 2.5. Measurement of the Diameter of Convoluted Tubules

The image is a measuring field of rat testicular section, on which two overlapping rectangular measuring boxes are superimposed. According to the distribution of the small tubules in the image, the small measuring box is used to select the spermatospermic tubules to be measured. It can be seen that according to the forbidden line rule, there are only two tubules A and B in the small measuring box, and the arrow line is the outer cut diameter of the two tubules [20]. 

As shown in Figure 1-II, the image is a measuring field of pig testicle section, on which two overlapping rectangular measuring boxes are superimposed. According to the distribution of small tubules in the image, the small measuring box is used to select the spermatogenous tubules to be measured. It can be seen that according to the forbidden line rule, there are only two tubules C and D in the small measuring box. The arrow line is the outer tangent diameter of the two tubules.

### 2.6. Protein Extraction and Detection

An amount of 0.2 g of cryptorchid and healthy testis tissue samples was weighed. In total, 1 mL RIPA and 10 μL PMSF of lysate were added onto them, respectively. The samples were placed in a shaker for 2.5 h, centrifuged at 4 °C for 12,000 r·min for 12 min, and the supernatant was absorbed for use. A 4× buffer was added onto the extracted protein samples proportionally and mixed by denaturation in a metal bath at 95 °C for 8 min. The prepared tissue samples were transferred from the gel to the PVDF membrane through 5% SDS-PAGE gel concentration and separation gel electrophoresis according to the random blocking principle (Millipore Corporation, Billerica, MA, USA). After quick sealing, incubation cross reaction was suitable for mouse monoclonal antibody VEGF (ab53465, abcam, 1:1000 dilution), rabbit polyclonal antibody EGFR (ab32198, abcam, 1: 1100 dilution), mouse monoclonal antibody P53 (ab154036, abcam, 1:1200 dilution), rabbit polyclonal antibody NF-κB (64921, Cell Signaling Technology (CST), Danvers, MA, USA, 1:900 dilution), overnight at 4 °C. The membrane with complete primary antibody incubation was washed with 1× Tris buffered saline Tween (TBST) 4 times (4 min/time) and reacted with HRP-labeled secondary antibody (1:1000 dilution) at room temperature for 1.5 h. After washing with 1× TBST 4 times (4 min/time), the imprinting of VEGF/EGFR and P53/NF-κB on the membrane was detected using ECL kit (NCM Biotech, Suzhou, China). Finally, photodensitometry (Bio-Rad, Hercules, CA, USA) was used to measure the optical density of the imprinted bands, with GAPDH as a reference.

### 2.7. Immunohistochemical Detection of VEGF/EGFR and P53/NF-κB

The cryptorchid tissue and healthy testicular tissue of Erhualian pigs fixed with 4% paraformaldehyde solution at room temperature could be preserved for a long time. Tissue blocks used for the test were embedded in paraffin, sliced, dried, and preserved at 4 °C.

To detect the positive immunohistochemical reactions of VEGF/EGFR and P53/NF-κB, all the prophase experimental steps were completed in strict accordance with the immunohistochemical procedure. They were treated with 3% deionized H_2_O_2_ for 15 to 20 min and sealed with goat serum for 15 to 20 min, added to monoclonal antibody VEGF (goat anti-mouse, 1:300, Bioss, Beijing, China), polyclonal antibody EGFR (goat anti-rabbit, 1:400, CST, USA), monoclonal antibody P53 (sheep against mouse, 1:500, Abcam, Boston, MA, USA), and polyclonal antibody NF-κB (goat anti-rabbit, 1:400, CST, USA), and incubated overnight. After washing, the corresponding secondary antibody was added, and tissues were incubated (Goat Anti-Mouse IgG (H+L)/HRP, bs-40296, bioss, diluted at 1:300; Goat Anti-Rabbit IgG (H+L)/HRP, bs-40295G, bioss, 1:300 dilution).

### 2.8. Immunofluorescent Detection of VEGF/EGFR and P53/NF-κB

For immunofluorescence staining, all operations prior to primary antibody incubation were identical to that of immunohistochemistry. The reaction-labeled samples were redyed with 3-3’-diaminobenzidine. Fluorescent secondary antibodies (Goat anti-Rabbit IgG (H+L), bs-40295G-IRDye8, Bioss, 1:600 dilution) were directly added onto samples instead of secondary antibodies for incubation for 2.5 h in immunofluorescence operation and then sealed for observation.

### 2.9. Data Statistics and Analysis

Image-Pro plus 6.0 software was used to measure the grayscale of optical density for the results of Western blot, immunohistochemistry, and immunofluorescence. SPSS21.0 software was used to calculate the experimental data, and the final results were expressed as mean ± SEM. The differences between samples were tested using independent sample t tests, and the differences between different groups of samples were analyzed using one-way ANOVA (LSD). Remarks: *p* < 0.01 means very significant difference; *p* < 0.05 means significant difference; and *p* > 0.05 indicates no significant difference.

## 3. Results

### 3.1. Observation of Special Staining of Cryptorchid Tissue

**Figure 2:**
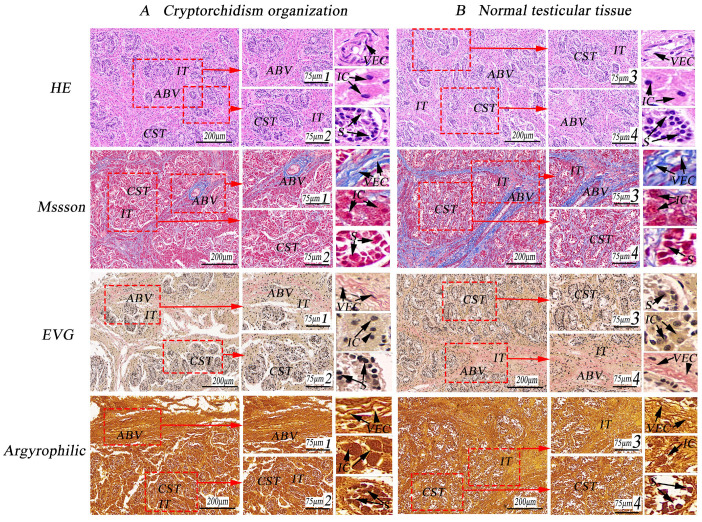
Special staining of porcine testicular tissue. HE/Masson/EVG/Argyrophilic: the histological characteristics of undescended testis and normal testis were observed using Masson staining. (**A**): Cryptorchidism tissue, 100×; 1 and 2 were the higher magnifications of A, 267×; (**B**): Normal testicular tissue, 100×; 3 and 4 were the local amplification of B, 267×; convoluted seminiferous tubules (CST); artery blood vessel (ABV); vascular endothelial cell (VEC); interstitial tissue (IT); collagenous fiber (CF); interstitial cells (IC); small vein (SV); myoid cells (MC); primary spermatocytes (PS); spermatogonia (S).

Following special histological staining with Masson and EVG, the cryptorchid tissue exhibited a scarcity of blue and red elastic fibers between the testicular lobules, accompanied by numerous scattered interstitial cells. The lumen contained many spermatogonial cells, with very few primary spermatocytes and absence of secondary spermatocytes, spermatid cells, and spermatozoa (as shown in Figure 2). By contrast, normal testes displayed a complete histological structure characterized by more blue and red elastic fibers between the testicular lobules, evenly distributed interstitial cells, well-developed convoluted seminiferous tubules, abundant blood vessels, a large number of spermatogonia, and a greater presence of primary spermatocytes within the tubules (as shown in Figure 2).

A total of 10 Erhualian pig testes, each stained in three replicates, were randomly observed and measured. Results from Argyrophilic staining revealed that compared to normally developing testes, the connective tissue, spermatogenic tubules, and blood vessels in cryptorchidism appeared brown and exhibited a darker stain. Conversely, normal testicular tissue displayed a lighter stain, accompanied by increased nuclear division of mesenchymal cells and spermatogenic cells (Figure 2).

### 3.2. Counting the Number of Convoluted Tubules

The measurements of convoluted seminiferous tubules and their diameter are detailed in Table 1, with scientific statistical analyses conducted using the calculation method specified in the cited reference (as indicated in Table 1). In three replicates per pig, the quantity of cryptorchid convoluted seminiferous tubules was significantly lower than that in normal testes, averaging 8.41 per/mm^2^ compared to the normal testes’ average of 14.21 per/mm^2^, demonstrating a significant difference (*p* < 0.01, as shown in Figure 2A). 

### 3.3. Measurement of the Diameter of Convoluted Tubules 

The diameter of cryptorchid convoluted seminiferous tubules was significantly smaller than that of normal testes, with an average diameter of 50.99 µm compared to the average diameter of 63.67 µm for the normal testes (as outlined in Table 2). The observed difference was statistically significant (*p* < 0.01, as shown in Figure 2B).

### 3.4. Analysis of VEGF/EGFR and P53/NF-κB Protein Expression

**Figure 3:**
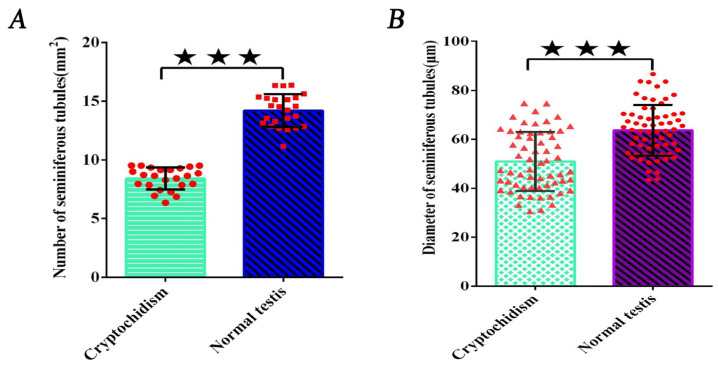
Measurement number and diameter of convoluted tubules. (**A**). Measurement of the number of convoluted tubules. (**B**). Measurement of the diameter of convoluted tubules. The five-pointed star represents differences, and the three five-pointed stars represent extremely significant differences (⋆⋆⋆ for *p* < 0.01).

**Figure 4:**
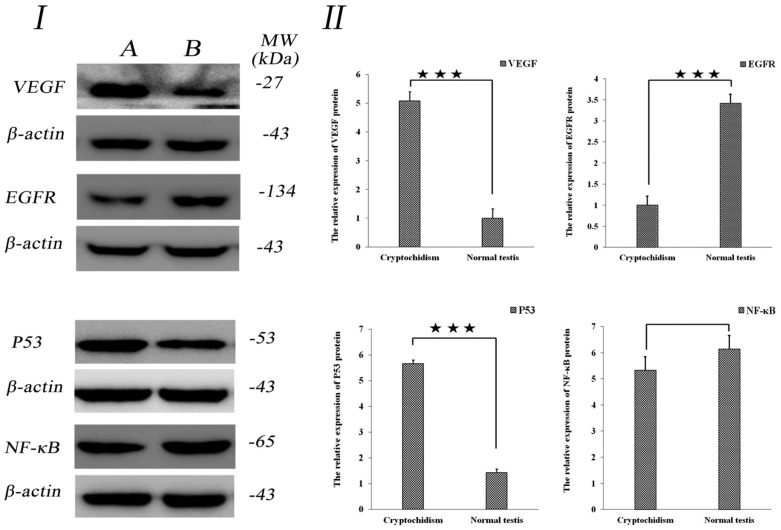
Protein expression in testicular tissue of Erhualian pigs. (**I**). Expression bands and molecular weight sizes of VEGF/EGFR and P53/NF-κB proteins. A is cryptorchid tissue and B is normal testicular tissue. (**II**). Different histogram represents expressions of VEGF/EGFR and P53/NF-κB in different testicular tissues from pigs. The five-pointed star represents differences, and the three five-pointed stars represent extremely significant differences (⋆⋆⋆ for *p* < 0.01).

No disparity was observed in the expression of VEGF/EGFR and P53/NF-κB proteins among cryptorchid tissues from different individuals. However, notable differences in expression were observed when comparing cryptorchid and normal testicle tissues (*p* < 0.01). Particularly, the expression of VEGF and P53 in cryptorchid tissues was significantly higher than in normal testicular tissue, while the expression of EGFR protein in normal testis tissues was significantly higher than in cryptorchid tissues (as demonstrated in Figure 3A), illustrating significant distinctions (*p* < 0.01). Conversely, NF-κB exhibited a high expression in both cryptorchid and normal testis tissues, with no significant difference observed (*p* > 0.05) (as depicted in Figure 4).

### 3.5. Immunohistochemical Expression Analysis of VEGF/EGFR and P53/NF-κB

**Figure 5:**
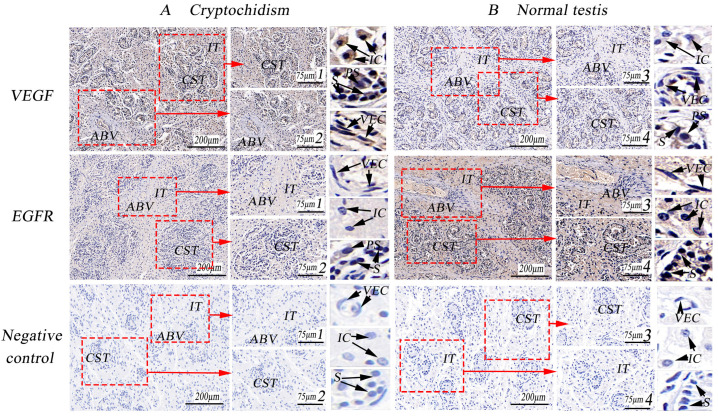
Immunohistochemical staining of VEGF and EGFR in porcine cryptorchidism and normal testis. (**A**). Cryptorchid tissue, 100×; 1 and 2 are local amplifications of A, 267×; (**B**). Normal testicular tissue, 100×; 3 and 4 are higher magnifications of B, 267×; CST. Convoluted seminiferous tubules (CST); artery blood vessel (ABV); vascular endothelial cell (VEC); interstitial tissue (IT); collagenous fiber (CF); interstitial cells (IC); small vein (SV); myoid cells (MC); primary spermatocytes (PS); spermatogonia (S).

Immunohistochemistry of cryptorchidism and normal testicular tissues revealed a stronger positive reaction of VEGF in the cytoplasm of cryptorchid interstitial cells and primary spermatocytes and a weaker expression in the cytoplasm of spermatogonial cells and in the nucleus. Similar staining patterns were observed in normal testicular tissues, although the expression was weaker. Conversely, EGFR exhibited weak expression in cryptorchid tissues, but it was strongly expressed in the cytoplasm of normal testicular mesenchymal cells, spermatogonia, and primary spermatocytes, with no expression in the nucleus. In addition, VEGF was strongly expressed in the blood vessels of cryptorchid tissues, while EGFR was strongly expressed in the blood vessels of normal testicular tissues (as depicted in Figure 5). Optical density analysis from immunohistochemistry revealed that VEGF expression in cryptorchid tissues was significantly higher than in normal testis, while the expression of EGFR was just the opposite, demonstrating distinct differences (as indicated in Table 3, *p* < 0.01).

**Figure 6:**
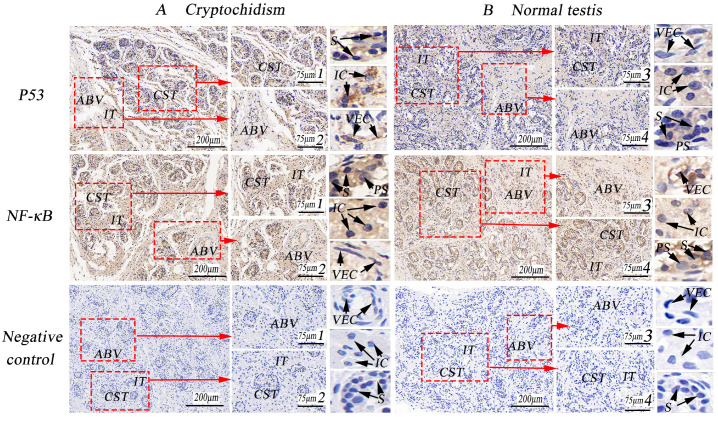
Immunohistochemical staining of P53 and NF-κB in porcine cryptorchidism and normal testis. (**A**). Cryptorchid tissue, 100×; 1 and 2 are local amplifications of A, 267×; (**B**). Normal testicular tissue, 100×; 3 and 4 are higher magnifications of B, 267×; convoluted seminiferous tubules (CST); artery blood vessel (ABV); vascular endothelial cell (VEC); interstitial tissue (IT); collagenous fiber (CF); interstitial cells (IC); small vein (SV); myoid cells (MC); primary spermatocytes (PS); spermatogonia (S).

In cryptorchid tissues, the positive reaction of P53 was robust in the cytoplasm of mesenchymal cells and spermatogonial cells, with weak expression in the nucleus. Conversely, the expression was weak in normal testicular tissues. The positive reaction of NF-κB was stronger in the cytoplasm of mesenchymal cells, spermatogonial cells, and primary spermatocytes in both cryptorchid and normal testicular tissues, with a weak positive reaction also observed in the nucleus. Additionally, P53 showed weak expression in blood vessels, while it exhibited strong positivity in the vascular wall of the testicular interstitial connective tissue where NF-κB was highly expressed (as illustrated in Figure 6). Immunohistochemical optical density analysis indicated that the expression of P53 in cryptorchid tissue was significantly higher than in normal testicle tissue, demonstrating a distinct difference (as indicated in Table 3, *p* < 0.01), whereas NF-κB was highly expressed.

### 3.6. Immunofluorescence Expression Analysis of VEGF/EGFR and P53/NF-κB

**Figure 7:**
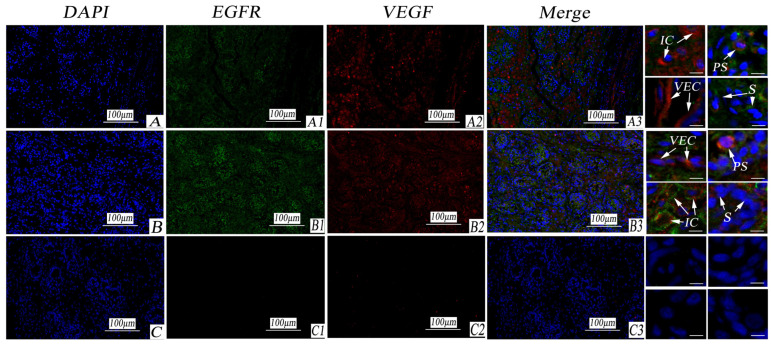
Immunofluorescence staining of VEGF and EGFR in porcine cryptorchidism and normal testis. (**A**). Cryptorchid tissue; (**A1**): EGFR positive marker; (**A2**): VEGF positive marker; (**A3**): EGFR and VEGF double fluorescent labeling. (**B**). Normal testicular tissue; (**B1**): EGFR positive marker; (**B2**): a positive marker for VEGF; (**B3**): double fluorescent labeling of EGFR and VEGF. (**C**). Negative control; (**C1**): EGFR negative control; (**C2**): VEGF negative control. (**C3**): a double-labeled negative control of EGFR and VEGF. Convoluted seminiferous tubules (CST); artery blood vessel (ABV); vascular endothelial cell (VEC); interstitial tissue (IT); collagenous fiber (CF); interstitial cells (IC); small vein (SV); myoid cells (MC); primary spermatocytes (PS); spermatogonia (S). Note: Blue stains the nucleus. Green is marked with EGFR protein staining. Red is marked with VEGF protein staining.

Immunofluorescence-positive reactions revealed that VEGF exhibited a strong positive reaction in the cytoplasm of cryptorchid mesenchymal cells and primary spermatocytes, with weaker expression in the cytoplasm of spermatogonia. In normal testicular tissues, the positive expression of mesenchymal cells and primary spermatocytes was strong, with weak expression observed in the nucleus. VEGF was prominently expressed in the blood vessels of testicular tissues. Conversely, EGFR was weakly expressed in cryptorchid tissue and exhibited slight positivity in the cytoplasm of spermatogonial cells. Its expression was stronger in the cell membrane of normal testicular mesenchymal cells, as well as in the cytoplasm and nucleus of primary spermatocytes. Meanwhile, Ig-G served as the negative control, revealing no positive reaction (as shown in Figure 7).

**Figure 8:**
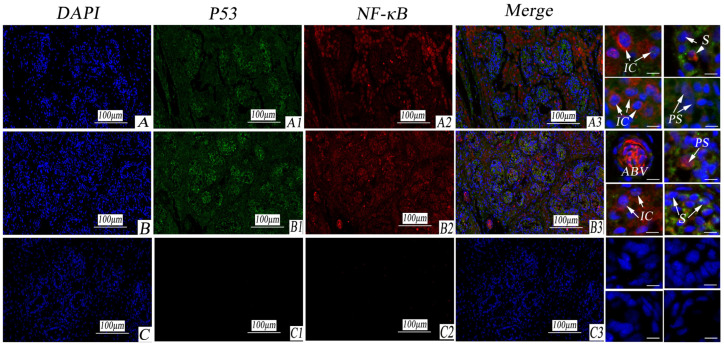
Immunofluorescence staining of P53 and NF-κB in porcine cryptorchidism and normal testis. (**A**). Cryptorchid tissue; (**A1**). P53 positive marker; (**A2**). NF-κB positive marker; (**A3**). EGFR and NF-κB double fluorescent labeling. (**B**). Normal testicular tissue; (**B1**). P53 positive marker; (**B2**). a positive marker for NF-κB. (**B3**). double fluorescent labeling of P53 and NF-κB. (**C**). Negative control; (**C1**). P53 negative control; (**C2**). NF-κB negative control. (**C3**). a double labeled negative control of P53 and NF-κB. Convoluted seminiferous tubules (CST); artery blood vessel (ABV); vascular endothelial cell (VEC); interstitial tissue (IT); collagenous fiber (CF); interstitial cells (IC); small vein (SV); myoid cells (MC); primary spermatocytes (PS); spermatogonia (S). Note: Blue stains the nucleus. Green is marked with P53 protein staining. Red is marked with NF-κB protein staining.

P53 exhibited strong expression in the cytoplasm of spermatogonial cells in both cryptorchid and normal testes but weak expression in the interstitial cells and blood vessels of the testis. NF-κB demonstrated strong expression in the cytoplasm of primary spermatocytes and mesenchymal cells in both cryptorchid and normal testes, with weak expression in spermatogonial cells. Moreover, NF-κB displayed strong expression in blood vessels. In the negative control, Ig-G was used, revealing no positive reaction (as illustrated in Figure 8).

## 4. Discussion

Dysplasia and metabolic abnormalities in animals contribute to testicular dysfunction or disappearance. In the case of cryptorchidism in goats, the development of blood vessels in testicular tissue is arrested, and the diameter of seminiferous tubules is smaller compared to that of normal testicular tissues, accompanied by abnormal Sertoli cell development [21]. The number of seminiferous epithelium cell layers and the thickness of seminiferous tubules in 12.5, 25, and 50 mg/L melamine treatment groups were significantly reduced. Simultaneously, the diameter of seminiferous tubules decreased in the 12.5 and 25 mg/L melamine-treated groups, and this was followed by a dramatic increase in the 50 mg/L melamine-treated group compared to controls [22]. Yuan Ligang et al. [23] reported that, compared with normal testicular interstitial structures, the spermatogenic tubules of cryptorchidism in Bactrian camels exhibited hypoplasia, sparse collagen fibers, a conspicuous distribution of reticular fibers, and weaker PAS- and AB- positive reactions in blood vessels and the membrana propria of spermatogenic tubules under a light microscope. Under an electron microscope, the basal membrane of the spermatogenic epithelium with respect to cryptorchidism showed marked hyperplasia, with fewer peripheral type I collagen fibers and atypical peritubular myoid cells; capillaries and peristomal fibers were commonly observed. 

In this experiment, observations via Masson and EVG staining revealed a reduced presence of blue and red elastic fibers in the lobules of cryptorchid tissues, along with numerous scattered interstitial cells. The lumen contained many spermatogonial cells; very few primary spermatocytes; and an absence of secondary spermatocytes, spermatocytes, and spermatozoa. In a study on the cryptorchid histology of companion Beagle dogs, Hu et al. found collagen fibers to be hyperplastic, with only a few spermatogonial cells and Sertoli cells present in convoluted seminiferous tubules. The epididymal interstitium was widened, containing numerous proliferating collagen fibers, and the epididymal duct lacked sperm [24]. In addition, during the induction of cryptorchidism in rats, germ cells in the convoluted seminiferous tubules of the abdominal testis exhibited degenerative changes, including spermatocytes and spermatozoa [25]. Argyrophilic staining in this experiment revealed that compared to normally developing testes, the connective tissues, spermatogenic tubules, and blood vessels in cryptorchidism appeared brown and exhibited a darker stain than that of normal testes. Conversely, normal testicular tissues were less stained, and there was an increase in the nuclear division of mesenchymal cells and spermatogenic cells. However, no histological observations of cryptorchidism in pigs have been reported to date. This experiment is the first to report on the structural abnormalities resulting from testicular dysplasia and metabolic disorders, providing an essential reference for clinical research on cryptorchidism in pigs.

**Figure 9:**
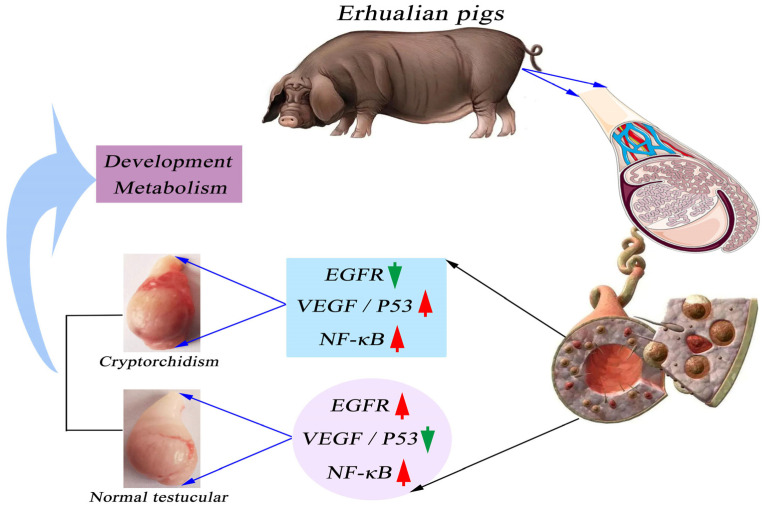
Schematic expression of VEGF/EGFR and P53/NF-κB in testicular tissue. The high expression of VEGF/P53 may be an important factor in the production of cryptorchidism. However, high expression of EGFR and NF-κB is the main factor to maintain normal testis development and metabolism. Note: The red shear head is high in protein expression. Green arrows are low in protein expression.

As the most crucial factor influencing cell growth and metabolism, the activity of VEGF directly affects the growth, development, and functional metabolism of target organs. Studies have shown that plasma and testicular microvasculature VEGF levels were elevated in the chronic intermittent hypoxia (CIH) group, with more evident testicular vessel distribution in CIH rats [26]. Mutant mice with seminiferous tubules exhibiting the overexpression of vascular endothelial growth factor (VEGF) displayed hypospermatogenesis, a marked reduction in the volume proportions of interstitial tissues and Leydig cells, a significant decrease in the diameter of seminiferous tubules and the height of their epithelium, vacuolation in the epithelium of the seminiferous tubules with the occurrence of mast cells, signs of delayed spermiation, and changes in sperm morphology [27]. The current study observed that the expression level of VEGF in cryptorchidism was significantly higher than that in normal testicular tissues, indicating a notable difference (as illustrated in Figure 9). Scientific evidence has confirmed that excessive estradiol can induce a significant increase in VEGF in mice, resulting in the abnormal proliferation of Leydig cells—a crucial inducing factor for testicular tumors [28]. In this study, VEGF was strongly expressed in the cytoplasm of Sertoli cells, while VEGFR-1 and VEGFR-2 were not expressed. VEGF collaborates with VEGFR-2 to regulate the proliferation of spermatogonial cells at the initial stage and participates in the VEGFR-1 regulation of spermatogenesis [29]. Androgen deficiency resulted in decreased sperm count and motility in rat testes, while microenergy treatment significantly reduced the stress response of the testes and improved the antioxidant capacity and antiapoptotic capacity [30]. This experiment revealed that VEGF was primarily expressed in mesenchymal cells with respect to cryptorchidism. Combining these findings with the aforementioned studies, it is speculated that the high expression of VEGF promotes abnormal proliferation and metabolic disorder of Leydig cells, serving as the main factor contributing to the occurrence of cryptorchidism.

EGFR is widely distributed on the surfaces of mammalian epithelial cells, fibroblasts, glial cells, and other cells, playing a crucial role in regulating cell growth, proliferation, differentiation, and physiological metabolism. It has been observed that epidermal growth factor receptor (EGFR) signaling is upregulated in hub cells after CySC ablation and reduced EGFR signaling inhibits the ability of testes to recover from ablation [31]. In the Leydig cells of rats, EGF (1 and 10 ng/mL) stimulates the proliferation of stem Leydig cells on the surface of convoluted seminiferous tubules and isolated CD90+ stem Leydig cells and progenitor Leydig cells, but it blocks their differentiation. Additionally, EGF exerts anti-apoptotic effects on progenitor Leydig cells [32]. This study revealed that the expression of EGFR in normal testes was higher than that in cryptorchid tissues, indicating a distinct difference (as shown in Figure 9). Research has shown that somatic CG6015 regulates CySC maintenance and GSC differentiation via EGFR signaling, inhibiting the aberrant activation of germline dpERK signals [33]. The testis-specific isoform of Na/K-ATPase interacts with caveolin-1, Src, epidermal growth factor receptor (EGFR), and extracellular signal-regulated kinase 1/2 (ERK1/2) in the raft and non-raft domains of the plasma membrane of bovine sperm during capacitation [34]. EGFR was highly expressed in the cytoplasm of normal Leydig cells, vascular endothelial cells, and spermatogonial cells. The aforementioned studies indicate that EGFR is involved in the growth and distribution of Leydig cells, the proliferation of vascular endothelial cells, and the development and metabolism of spermatogonial cells, and it contributes to maintaining the normal physiological function of testes.

The P53-mediated cell signal transduction pathway is crucial in regulating normal cell life activities. Significantly increased apoptosis levels were observed in male testes, leading to the increased expression of P53-mediated apoptotic pathways. Concurrently, histological alterations, such as a substantial decrease in the thickness of the testis basement membrane, were also observed [35]. These findings collectively indicate the involvement of the P53 signaling pathway in DEHP-induced prepubertal testicular injury by promoting cell apoptosis and inhibiting the cell proliferation of Leydig cells [36]. The current study revealed that the expression of P53 in cryptorchid tissues was higher than that in normal testicular tissues, and the difference was significant (as shown in Figure 9). In As-exposed mice, mature seminiferous tubules and epididymal sperm count were reduced. The cell growth index decreased in As-exposed GC-1 cells, and flow analysis showed that As-exposed GC-1 cells were arrested at the G2/M phase. NAC alleviated As-evoked DNA damage, genotoxic stress, cell proliferation inhibition, and sperm count reduction [37]. P53 was mainly expressed in the cytoplasm of Leydig cells and spermatogonial cells in cryptorchid tissues but not in the nucleus, while it was weakly expressed in normal testicular tissues. Therefore, as an important apoptotic gene, it is speculated, in conjunction with the studies, that cryptorchid Leydig cells proliferate extensively and are arranged in a disorderly manner during the early stage. During late cryptorchidism, a substantial number of apoptosis events and the arrested development of Leydig cells occur, resulting in a small volume of cryptorchidism.

NF-κB is implicated in the functional metabolism of cells in response to external stimuli, such as cytokines, radiation, heavy metals, and viruses. Studies have demonstrated that a decrease in the mRNA expression of antioxidant enzymes, increases in free radical products, the upregulation of mRNA, and the protein expression of nuclear factor-κB and proinflammatory cytokines, and the inhibition of mRNA and the protein expression of testosterone synthases result in decreases in serum testosterone levels and sperm quality and an increase in sperm apoptosis [38]. In this experiment, the high levels of NF-κB expression with respect to cryptorchidism indicates that the apoptosis of Leydig cells and spermatogonial cells in cryptorchid tissues is a crucial factor that results in dysplasia with respect to cryptorchidism (as shown in Figure 9). Zheng et al. reported that in porcine testicular cells infected with the Japanese encephalitis virus, the expression of proinflammatory factors was notably reduced after RIG-I knockout or NF-κB-specific inhibitor treatment, and the metabolic activity of NF-κB in porcine testicular cells with RIG-I knockout was significantly inhibited [39]. Huynh et al. revealed that NF-κB could be involved in cocaine-induced reproductive dysfunction and the abnormal metabolism of glutathione peroxidase (GPx)-1 in male mice. Moreover, the mRNA levels of Nrf2 and NF-κB in testicular tissues of rats with lead poisoning were notably increased, serving as an important index for determining the levels of androgen and sperm quality in serum [40,41]. Notably, NF-κB is also highly expressed in normal testicular tissues in our experiments. Combining this with the above literature, it is believed that the high expression of NF-κB maintains the division and proliferation of testicular Leydig cells during the developmental stage, promoting spermatogonial cells’ transition to mature sperm and supporting the normal physiological metabolism and function of the testis.

## 5. Conclusions

Significant differences exist in tissue morphology and structure between undescended testes and normal testes. The metabolic expression of VEGF and P53 in cryptorchidism is significantly higher than in normal testes. This suggests their involvement in the metabolism and growth distribution of testicular Leydig cells, as well as the development of convoluted seminiferous tubules and the maturation of spermatogenic cells. These findings underscore the significance of VEGF and P53 as key factors inducing the occurrence of cryptorchidism in pigs.

## Figures and Tables

**Table 1:** Average number of tubules per pig and population mean.

Number	Cryptochidism (Number/mm^2^)	Normal Testis (Number/mm^2^)
Sample 1	8.62 ± 0.71	13.74 ± 1.04
Sample 2	7.87 ± 1.14	14.58 ± 0.31
Sample 3	8.89 ± 0.81	13.36 ± 0.83
Sample 4	7.66 ± 0.57	15.11 ± 0.61
Sample 5	8.54 ± 1.05	14.07 ± 1.24
**Mean value**	**8.31** ± **0.86** **^A^**	**14.17** ± **0.81** **^B^**

Table note: A and B show significant differences of mean values, *p* < 0.01. Mean value (Average number of tubules per pig and population mean).

**Table 2:** Tubule diameter and total mean value of each pig.

Diameter	Cryptochidism (μm)	Normal Testis (μm)
Sample 1	47.03 ± 6.02	71.13 ± 7.31
Sample 2	42.83 ± 2.79	60.23 ± 3.35
Sample 3	55.01 ± 3.97	61.33 ± 8.51
Sample 4	57.87 ± 2.13	62.08 ± 4.11
Sample 5	47.77 ± 3.65	62.49 ± 5.87
**Mean value**	**50.11** ± **3.71** **^A^**	**63.45** ± **5.83** **^B^**

Table note: A and B show significant differences of mean values, *p* < 0.01. Mean value (Tubule diameter and total mean value of each pig).

**Table 3:** Integrated optical density of VEGF/EGFR and P53/NF-κB proteins in different tissues.

	Integral Optical Density (IntDen/Area)	
	Cryptochidism	Normal Testis
VEGF	3.0754 ± 0.3725 ^A^	1.1502 ± 0.3254 ^B^
EGFR	1.1682 ± 0.2148 ^A^	3.4133 ± 0.4945 ^B^
P53	3.6564 ± 0.3127 ^A^	1.4251 ± 0.1347 ^B^
NF-κB	4.7085 ± 0.5241 ^A^	5.1317 ± 0.3124 ^A^

Table Note: A and B show significant differences of mean values.

## Data Availability

Data are contained within the article.
